# Multilayer-omics analyses of human cancers: exploration of biomarkers and drug targets based on the activities of the International Human Epigenome Consortium

**DOI:** 10.3389/fgene.2014.00024

**Published:** 2014-02-14

**Authors:** Yae Kanai, Eri Arai

**Affiliations:** ^1^Division of Molecular Pathology, National Cancer Center Research InstituteTokyo, Japan; ^2^Core Research for Evolutional Science and Technology, Japan Science and Technology AgencyTokyo, Japan

**Keywords:** epigenetics, epigenome, DNA methylation, International Human Epigenome Consortium (IHEC), multilayer/integrated disease omics analyses

## Abstract

Epigenetic alterations consisting mainly of DNA methylation alterations and histone modification alterations are frequently observed in cancers associated with chronic inflammation and/or persistent infection with viruses or other pathogenic microorganisms, or with cigarette smoking. Accumulating evidence suggests that alterations of DNA methylation are involved even in the early and precancerous stages. On the other hand, in patients with cancers, aberrant DNA methylation is frequently associated with tumor aggressiveness and poor patient outcome. Recently, epigenome alterations have been attracting a great deal of attention from researchers who are focusing on not only cancers but also neuronal, immune and metabolic disorders. In order to accurately identify disease-specific epigenome profiles that could be potentially applicable for disease prevention, diagnosis and therapy, strict comparison with standard epigenome profiles of normal tissues is indispensable. However, epigenome mechanisms show heterogeneity among tissues and cell lineages. Therefore, it is not easy to obtain a comprehensive picture of standard epigenome profiles of normal tissues. In 2010, the International Human Epigenome Consortium (IHEC) was established to coordinate the production of reference maps of human epigenomes for key cellular states. In order to gain substantial coverage of the human epigenome, the IHEC has set an ambitious goal to decipher at least 1000 epigenomes within the next 7–10 years. We consider that pathway analysis using genes showing multilayer-omics abnormalities, including genome, epigenome, transcriptome, proteome and metabolome abnormalities, may be useful for elucidating the molecular background of pathogenesis and for exploring possible therapeutic targets for each disease.

## MICRO RNAs AND HUMAN DISEASES

The Encyclopedia of DNA Elements (ENCODE) Consortium^1^ data have revealed in more detail the high degree of complexity of the mammalian transcriptome: 75% of the genome is transcribed into different types of RNA molecules, e.g., protein-coding, long non-coding, pseudogenes, and small RNA genes (Djebali et al., 2012). RNA molecules show much greater variety than previously suspected. Among such RNA molecules, microRNAs (miRNAs) are non-coding RNAs comprising about 22 nucleotides initially transcribed by RNA polymerase II as primary miRNA (pri-miRNA) molecule precursors that possess a stem loop structure (Jinek and Doudna, 2009). RNase III Drosha acts over pri-mRNAs generating a pre-miRNA containing the hairpin (Jinek and Doudna, 2009). The pre-miRNAs are then exported to the cytoplasm and processed by Dicer into mature miRNAs, which are subsequently translocated into the RNA-induced silencing complex (RISC; Gomes et al., 2013). Each miRNA has multiple tasks, such as transcriptional repression via binding to partially complementary sequences in the 3′-untranslated regions of the target mRNAs and direct mRNA degradation via binding to perfectly complementary sequences (He and Hannon, 2004). Therefore, deregulation of miRNA levels may disturb the expression profiles in cells, thereby playing a key role in induction of diseases, such as cancers, neurodegenerative diseases, and autoimmune diseases.

## EPIGENETICS AND miRNAs

Saito et al. revealed that treatment with the DNA demethylating agent 5-aza-2′-deoxycytidine and the histone deacetylase inhibitor 4-phenylbutyric acid induced marked changes in the expression profiles of miRNAs in human cancer cell lines. In particular, DNA hypermethylation and induction of active histone marks in the promoter region of miR-127 resulted in decreased and increased expression of miR-127, respectively (Saito et al., 2006). Activation of miR-512-5p by epigenetic treatment induced apoptosis of human gastric cancer cell lines via suppression of the *MCL1* gene (Saito et al., 2009). In the human colon cancer cell line HCT116, disturbance of miRNA expression patterns has been reported after disruption of both *DNA methyltransferase *(*DNMT*)* 1* and *DNMT3B* (Lujambio et al., 2007). Findings accumulated to date clearly indicate that expression levels of multiple miRNAs, such as let-7a-3, miR-1, miR-9-1, miR-9-3, miR-34a, mir34a*, mir34b/c, miR-124a, miR-126. miR127, miR-342, and miR-512-5p, are regulated epigenetically (Saito et al., 2013).

On the other hand, the expression of many proteins involved in epigenetics is regulated by miRNAs. For example, miR-152 acts as a tumor suppressor via suppression of *DNMT1* (Huang et al., 2010). The miR-29 family targets *DNMT3A* and *DNMT3B*, whereas miR-101 targets *EZH2* and may alter global chromatin structure (Fabbri et al., 2007). In addition, it has been shown that miRNA has the capacity to recognize chromatin by increasing the methylation of histone, for example through histone H3 lysine 27 tri-methylation (Kim et al., 2008). Thus the close connection between epigenetic alterations and miRNA dysregulation may have a great impact on human diseases.

## PARTICIPATION OF EPIGENETIC ALTERATIONS IN MULTISTAGE HUMAN CARCINOGENESIS

Epigenetic alterations, consisting mainly of DNA methylation alterations and histone modification alterations, are often observed in cancers that are associated with chronic inflammation and/or persistent infection with viruses, such as hepatitis B or C viruses, Epstein–Barr virus, and human papillomavirus, or with cigarette smoking (Kanai and Hirohashi, 2007). Accumulating evidence suggests that alterations of DNA methylation are involved even in the early and precancerous stages (Arai and Kanai, 2010). On the other hand, in patients with cancers, aberrant DNA methylation is frequently associated with tumor aggressiveness and poor patient outcome (Kanai, 2008). Precancerous conditions showing alterations of DNA methylation may progress rapidly and generate more malignant cancers (Kanai, 2010).

As we described in the webpage of our laboratory^2^, even though genetic alterations, such as activation of oncogenes and inactivation of tumor suppressor genes, have been considered to provide the molecular framework of multistage human carcinogenesis, genetic events alone may not explain the histological heterogeneity underlying the complex biological characteristics of tumors. Therefore, in the 1990s, we began to focus on epigenetic events that can be reversible, in an attempt to explain why cancers show such histopathological heterogeneity. At a time when only two genes, *RB* and *VHL*, were known as tumor suppressor genes silenced by DNA methylation, we showed for the first time that the *CDH1 *gene, which encodes the E-cadherin cell adhesion molecule and acts as tumor suppressor, is silenced by DNA methylation around the promoter region in human cancers (Yoshiura et al., 1995). In 1996, we demonstrated that DNA methylation alterations frequently occurred at multiple loci on chromosome 16, one of the hot spots for loss of heterozygosity in liver cancers. This preceded loss of heterozygosity even at the chronic hepatitis or liver cirrhosis stages, which are widely considered to be precancerous conditions. This was one of the earliest reports of aberrant DNA methylation at the precancerous stage (Kanai et al., 1996).

Since then, we have reported DNA methylation alterations in tissue specimens at precancerous stages and in cancers using a candidate-gene approach. As an example of inflammation-associated carcinogenesis, ductal adenocarcinomas of the pancreas frequently develop in a background of chronic pancreatitis. Under these conditions, at least a proportion of peripheral pancreatic duct epithelia may be at the precancerous stage. It has been reported that the average number of methylated tumor-related genes, the incidence of DNA methylation of at least one of such genes, and the expression level of *DNMT1* protein are increased in pancreatic duct epithelia with an inflammatory background, and in another precancerous lesion, pancreatic intraductal neoplasia (PanIN), in comparison with normal pancreatic duct epithelia (Peng et al., 2006).

Urothelial carcinomas of the urinary bladder, renal pelvis, and ureter are clinically remarkable because of their multicentricity and tendency to recur. Such multiplicity may be attributable to the “field effect.” Even non-cancerous urothelia showing no marked histological findings from patients with urothelial carcinomas can be considered precancerous, because they may have been exposed to carcinogens in the urine. It has been reported that the average number of methylated tumor-related genes and the expression level of *DNMT1* protein are increased in non-cancerous urothelia showing no marked histological findings from patients with urothelial carcinomas, in comparison with normal urothelia from patients without urothelial carcinomas (Nakagawa et al., 2005). Thus, overexpression of the major DNMT, *DNMT1*, may result in accumulated hypermethylation of DNA for tumor-related genes (Etoh et al., 2004). On the other hand, splicing alteration of *DNMT3B* may induce chromosomal instability through DNA hypomethylation of pericentromeric satellite regions (Saito et al., 2002).

As we described in the webpage of our laboratory^2^, after genome-wide epigenetic (epigenome) analysis had become practical, we employed the bacterial artificial chromosome array-based methylated CpG island amplification (BAMCA) method for overviewing the DNA methylation tendency of large individual chromosomal regions. Although precancerous conditions in the kidney have rarely been described, despite the lack of any marked histological findings or association with chronic inflammation or persistent infection with pathogens, it can be considered that non-cancerous renal cortex tissue obtained from patients with renal cancers is already at the precancerous stage showing genome-wide DNA methylation alterations (Arai et al., 2006). We showed that DNA methylation profiles at the precancerous stage are inherited by renal cancers developing in individual patients, and that DNA methylation alterations at the precancerous stage determine both the aggressiveness of subsequently developing cancers and patient outcome through inducing further epigenetic and genetic alterations (Arai et al., 2009a). In addition, we have developed indicators for carcinogenetic risk estimation in patients with chronic hepatitis and liver cirrhosis (Arai et al., 2009b), indicators for estimating the risk of development of urothelial carcinomas that can be determined from urine samples (Nishiyama et al., 2010), diagnostic markers of pancreatic cancer that can be assessed from specimens of pancreatic juice (Gotoh et al., 2011), and indicators for prognostication of kidney, liver, pancreas, and urinary bladder cancers based on DNA methylation profiling. Based on these findings, we have filed patent applications for epigenome diagnosis techniques, and are now attempting to apply them practically.

## ACTIVITIES OF THE INTERNATIONAL HUMAN EPIGENOME CONSORTIUM (IHEC)

Recently, epigenome alterations have been attracting a great deal of attention from researchers who are focusing on not only cancers but also neuronal, immune, and metabolic disorders. On the basis of epigenome profiling, attempts are now being made to elucidate the molecular pathogenesis of such diseases and to explore possible biomarkers and drug targets. In order to accurately identify such disease-specific epigenome profiles that could be potentially applicable for disease prevention, diagnosis, and therapy, strict comparison with standard epigenome profiles of normal tissues is indispensable. However, epigenome mechanisms show heterogeneity among tissues and cell lineages. Therefore, it is not easy to obtain a comprehensive picture of standard epigenome profiles of normal tissues. Based on improvements in next-generation sequencing technology, international collaboration will likely help to reveal standard epigenome profiles.

In 2010, the IHEC was established by researchers and founding agencies from Canada, South Korea, the EU, Italy, Germany, Japan, and the USA (Bae, 2013). As described in the webpage of IHEC^3^, the primary goal of the IHEC is “to coordinate the production of reference maps of human epigenomes for key cellular states that are relevant to health and diseases.” In order to achieve substantial coverage of the human epigenome, the IHEC has set an ambitious goal to decipher at least 1000 epigenomes^3^. To attain this goal, IHEC will use robust techniques to generate (1) high-resolution maps of histone modifications, H3K4me3, H3K9me3, H3K27me3, H3K27ac, H3K4me1, and H3K36me3, (2) high-resolution DNA methylation maps, (3) landmark maps of transcription start sites for all protein-encoding genes, and (4) a comprehensive catalog of non-coding and small RNAs and their patterns of expression^3^. The target cell types being studied by each team in the participating countries are shown on the IHEC website^4^.

**FIGURE 1:**
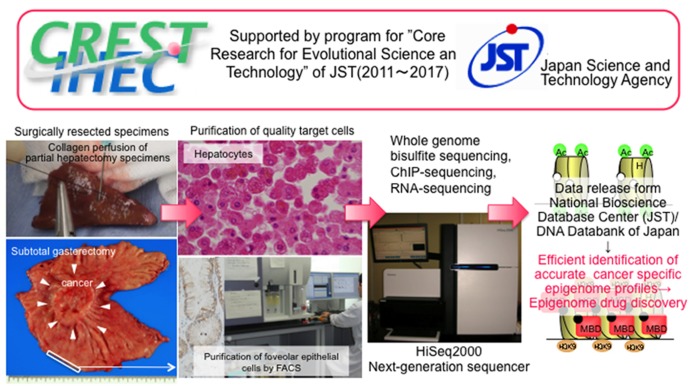
**Activity of our team, one of the Japan Teams of the Inter-national Human Epigenome Consortium supported by the Core Research for Evolutional Science and Technology division of the Japan Science and Technology Agency (modified from the webpage of our laboratory [http://www.ncc.go.jp/en/nccri/divisions/01path/01path01.html]).** We are now performing whole-genome bisulfite sequencing using the post-bisulfite adaptor-tagging method, chromatin immunoprecipitation-sequencing, and RNA-sequencing of purified target cells, i.e., hepatocytes and other live cell lineages, foveolar epithelial cells, and other gastric epithelial cell lineages, and absorptive epithelial cells from the ascending and descending colon and rectum. Accurate epigenome profiling of normal cells will allow the identification of disease-specific epigenome profiles, thus facilitating a potential breakthrough in the prevention, diagnosis, and therapy of diseases.

In Japan, three Japanese IHEC teams^5^ including our team are supported by the Core Research for Evolutional Science and Technology division of the Japan Science and Technology Agency. To strengthen the research bases for cancers of digestive organs, including hepatocellular carcinomas and gastric carcinomas, which show high incidences in Japan, we are now performing standard epigenome analyses of normal epithelial cell lineages in digestive organs (**Figure 1**). Target cells of sufficient quality and quantity are being obtained from materials surgically resected from a range of Japanese patients. For example, for liver, we have obtained samples of normal liver tissue distant from sites of liver metastases from primary colon cancers in partial hepatectomy specimens from patients without viral hepatitis, chronic hepatitis, or liver cirrhosis. To isolate hepatocytes, we have performed collagenase perfusion of cannulated branches of the hepatic vein, followed by low-velocity centrifugation. On average, more than 10^7^ dispersed cells can be obtained from each case, and immunocytochemistry has confirmed that the hepatocytes are more than 95% pure. In the stomach and colorectum, we initially employ the crypt isolation technique****and collagenase digestion. Thereafter, each normal cell lineage is purified by fluorescence activated cell sorting using appropriate antibodies.

Members of our IHEC team have originally developed the post-bisulfite adaptor-tagging method (PBAT), which is an efficient library preparation method for whole-genome bisulfite sequencing (Miura et al., 2012). For the PBAT method, we first perform bisulfite modification followed by adaptor ligation using random priming. The PBAT method minimally requires sub-microgram DNA for mammalian whole-genome bisulfite sequencing without global PCR amplification. A good correlation of the DNA methylation pattern was observed among PBAT, the standard Methyl C-seq methodology developed by Lister et al. (2008), and the Illumina beads chip Infinium assay. The PBAT method is advantageous in that it requires only a small amount of genomic DNA but has good coverage of GC-rich regions, especially in CpG islands and gene-rich chromosomes. We now propose to make the PBAT method one of the standard protocols for IHEC. Under the supervision of the IHEC, we intend to disclose the data we obtain through the National Bioscience Database Center supported by the Japan Science and Technology Agency. Accurate standard epigenome profiles of digestive organ epithelial cells obtained through IHEC activities will be used to explore more useful biomarkers and drug targets of digestive organ cancers.

## MULTILAYER/INTEGRATIVE DISEASE OMICS ANALYSES FOR EXPLORATION OF BIOMARKERS AND DRUG TARGETS

**FIGURE 2:**
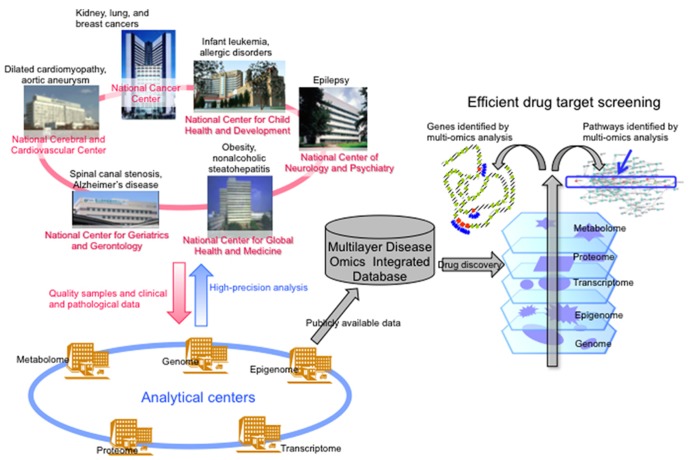
**Brief overview of the “Comprehensive exploration of drug targets based on multilayer/integrative disease omics analyses” project supported by the Program for Promotion of Fundamental Studies in Health Sciences of the National Institute of Biomedical Innovation (modified from the webpage of our laboratory [http://www.ncc.go.jp/en/nccri/divisions/01path/01path01.html]).** In this project, researchers in several National Centers in Japan have been split up into analytical centers for genome, epigenome, transcriptome, proteome, and metabolome. We have been focusing on molecules or molecular pathways that are impaired by multiple mechanisms, such as genome and epigenome events, or events in all five omics layers, which may participate in the molecular pathogeneses of those diseases and might become drug targets.

Recently big data analysis has impacted various fields of bioscience, especially disease research. It may not be appropriate to perform epigenome analysis including miRNA analysis using clinical samples. Instead, simultaneous multilayer/integrative disease omics analyses would seem more appropriate, including genome, epigenome, transcriptome, proteome, and metabolome analyses for exploration of drug targets. Since 2010, researchers at six National Centers in Japan, i.e., the National Cancer Center, National Cerebral and Cardiovascular Center, National Center for Neurology and Psychiatry, National Center for Global Health and Medicine, National Center for Child Health and Development and National Center for Geriatrics and Gerontology, have been engaged in a research project “Comprehensive exploration of drug targets based on multilayer/integrative disease omics analyses” supported by the Program for Promotion of Fundamental Studies in Health Sciences of the National Institute of Biomedical Innovation (NiBio) (**Figure 2**). This project has been divided among a number of centers specializing in genome, epigenome, transcriptome, proteome, and metabolome analyses of tissue specimens from patients with various diseases that show a high incidence in the Japanese population. Tissue and body fluid specimens, cultured cells and animal models of adult cancers, infant leukemia, allergic disease, dilated cardiomyopathy, aortic aneurysm, epilepsy, obesity, non-alcoholic steatohepatitis, spinal canal stenosis, and Alzheimer’s disease have been subjected to multilayer-omics analyses. As we described in the webpage of our laboratory^2^, we are especially focusing on molecules or molecular pathways which are impaired as a result of multiple mechanisms, such as events in all five omics layers, which may participate in the molecular pathogeneses of diseases and might become potential biomarkers and/or druggable targets (**Figure 2**).

With regard to epigenome analysis of adult cancers in this research project, 414 lung tissue specimens including normal lung tissue (LC) obtained from patients without any primary lung tumor, non-cancerous lung tissue (LN) obtained from patients with lung adenocarcinomas, and lung adenocarcinoma tissue (LT) itself have been subjected to single-CpG resolution Infinium assay. DNA methylation alterations on many probes were evident in LN samples relative to LC samples, and were inherited by, or strengthened in, LT samples. Unsupervised hierarchical clustering using DNA methylation levels in LN samples subclustered patients into clusters I, II, and III. Lung adenocarcinomas in cluster I developed from an inflammatory background in chronic obstructive pulmonary disease (COPD) in heavy smokers, and were locally invasive. Most patients in cluster II were non-smokers and had a favorable outcome. Lung adenocarcinomas in cluster III were most aggressive cancers in light smokers that developed before accumulation of the long-term effects of cigarette smoking, and were probably due to the direct actions of carcinogens, rather than the effects of inflammation. DNA methylation profiles reflecting carcinogenetic factors such as smoking and COPD appear to be established in LNs and may determine the aggressiveness of tumors developing in individual patients, and thus patient outcome (Sato et al., 2014). Among the genes for which DNA methylation status in LN samples was significantly correlated with recurrence of lung adenocarcinomas in individual patients, we focused on *ADCY5, EVX1*, and other genes that were involved in apoptosis and cell adhesion. The mRNA expression levels of these genes were directly regulated by DNA methylation, and a decrease of their mRNA expression in LT samples was significantly correlated with tumor aggressiveness (Sato et al., 2013). When these genes were ectopically expressed in lung cancer cell lines, growth suppression, and apoptosis were induced, indicating that these genes could become therapeutic targets of lung adenocarcinomas.

With regard to epigenome analysis during renal carcinogenesis, 245 renal tissue specimens including normal renal cortex tissue (RC) obtained from patients without any primary renal cancer, non-cancerous renal cortex tissue (RN) obtained from patients with clear cell renal cell carcinomas, and clear cell renal cell carcinoma tissue (RT) itself were subjected to the Infinium assay. DNA methylation levels at multiple Infinium probe sites were already altered in RN samples relative to RC samples. Unsupervised hierarchical clustering analysis based on DNA methylation levels at the CpG sites where DNA methylation alterations had occurred even in RN samples and were inherited by, and strengthened in, RT samples divided the clear cell renal cell carcinomas into CpG island methylator phenotype (CIMP)-positive and -negative clusters (Arai et al., 2012). Clinicopathologically aggressive cancers were accumulated in the CIMP-positive cluster, where the cancer-free and overall survival rates of the patients were significantly lower than in the CIMP-negative cluster. *FAM150A, GRM6, ZNF540, ZFP42, ZNF154, RIMS4, PCDHAC1, KHDRBS2, ASCL2, KCNQ1, PRAC, WNT3A, TRH, FAM78A, ZNF671, SLC13A5*, and *NKX6-2* have been identified as renal cell carcinoma-specific CIMP marker genes (Arai et al., 2012). Since CIMP-positive renal cell carcinomas show tumor aggressiveness and poorer patient outcome, we established criteria for prognostication of patients with clear cell renal cell carcinomas using renal cell carcinoma-specific CIMP marker genes. We are now performing pathway analysis based on a Bayesian estimation model using multiple genes showing frequent mutations and alterations of expression at the mRNA, miRNA, and protein levels based on multilayer-omics analyses in each of the CIMP-negative and CIMP-positive renal cell carcinomas for exploration of possible drug targets.

## PERSPECTIVES

Once DNA methylation alterations occur during multistage carcinogenesis, such alterations are stably preserved on DNA double strands through maintenance methylation mechanisms by *DNMT1*. Therefore, stable stratification of cancers reflecting clinicopathological diversity may be possible based on epigenome profiling. Genes showing epigenome alterations, such as CIMP-marker genes, may become excellent biomarkers discriminating each tumor type stratified on the basis of epigenome profiling. We consider that pathway analysis using genes showing multilayer-omics abnormalities after stratification based on epigenome profiling may be useful for elucidating the molecular background of carcinogenetic pathways and for exploring possible therapeutic targets for each tumor type.

## Conflict of Interest Statement

The authors declare that the research was conducted in the absence of any commercial or financial relationships that could be construed as a potential conflict of interest.

## Footnotes

1 https://genome.ucsc.edu/ENCODE/

2 http://www.ncc.go.jp/en/nccri/divisions/01path/01path01.html

3 http://ihec-epigenomes.org/about/objectives/

4 http://ihec-epigenomes.org/research/cell-types/

5 http://crest-ihec.jp/

## Abbreviations

CIMP: CpG island methylator phenotype;
COPD: chronic obstructive pulmonary disease;
IHEC: International Human Epigenome Consortium;
LC: normal lung tissue;
LN: non-cancerous lung tissue obtained from patients with lung adenocarcinoma;
LT: lung adenocarcinoma tissue;
PBAT: post-bisulfite adaptor-tagging;
RC: normal renal cortex tissue;
RN: non-cancerous renal cortex tissue obtained from patients with clear cell renal cell carcinoma;
RT: clear cell renal cell carcinoma tissue.
